# The role of mini-bronchoalveolar lavage fluid in the diagnosis of pulmonary tuberculosis in critically ill patients

**DOI:** 10.1186/s12879-020-04954-3

**Published:** 2020-03-18

**Authors:** Cynthia Pessoa Neves, Allyson Guimarães Costa, Izabella Picinin Safe, Alexandra de Souza Brito, Jaquelane Silva Jesus, Afranio Lineu Kritski, Marcus Vinicius Guimarães Lacerda, Miguel Viveiros, Marcelo Cordeiro-Santos

**Affiliations:** 1 Programa de Pós-Graduação em Medicina Tropical, Universidade do Estado do Amazonas (UEA), Manaus, AM Brazil; 2 Instituto de Pesquisa Clínica Carlos Borborema, Fundação de Medicina Tropical Dr. Heitor Vieira Dourado (FMT-HVD), Manaus, AM Brazil; 3 Diretoria de Ensino e Pesquisa, Fundação Hospitalar de Hematologia e Hemoterapia do Amazonas (HEMOAM), Manaus, AM Brazil; 4 Programa de Pós-Graduação em Imunologia Básica e Aplicada, Universidade Federal do Amazonas (UFAM), Manaus, AM Brazil; 5 Faculdade de Medicina, Universidade Federal do Rio de Janeiro (UFRJ), Manaus, AM Brazil; 6 Instituto Leônidas e Maria Deane, Fundação Oswaldo Cruz Amazônia, Manaus, AM Brazil; 7 Instituto de Higiene e Medicina Tropical, Universidade Nova de Lisboa, Lisbon, Portugal

**Keywords:** Pulmonary tuberculosis, Intensive care medicine, Diagnosis, Mini-BAL

## Abstract

**Background:**

The detection of *Mycobacterium tuberculosis* (MTB) in the intensive care unit (ICU) presents several challenges, mainly associated to the clinical state of the patient. The presence of HIV infection further aggravates this scenario, requiring a reliable collection method, with better performance in the microbiological/molecular techniques to be used. We evaluated the performance of two methods for sample collection, mini bronchoalveolar lavage (Mini-BAL) and endotracheal aspirate (ETA), for diagnosis of pulmonary tuberculosis (PTB) in critically ill patients.

**Methods:**

This prospective study involved 26 HIV positive ICU internalized patients, with presumptive PTB who required mechanical ventilation. Two samples were obtained prospectively from 26 HIV ICU patients with presumptive PTB by Mini-BAL and ETA. The samples were processed for smear microscopy, Löwenstein-Jensen medium and the BACTEC Mycobacteria Growth Indicator Tube 960 system®. We define as confirmed PTB patients with positive MTB culture. Furthermore, all samples obtained through the Mini-BAL were analyzed by Xpert® MTB/RIF.

**Results:**

Our results demonstrated that the respiratory samples obtained by Mini-BAL were able to increase MTB detection in critically ill patients with presumptive PTB. The Mini-BAL allowed 30% increased recovery and guaranteed enough sample volume for processing in all methods. In addition, the larger volume of the samples obtained with this technique enabled the Xpert® MTB/RIF molecular test for diagnosis of TB.

**Conclusions:**

The Mini-BAL showed be an acceptable alternative to ETA in this population, since these critically ill and often-immunocompromised patients are more likely to develop complications related to invasive procedures.

## Background

An increasing proportion (3–16%) of patients diagnosed with active tuberculosis (TB) require intensive care [1–3]. In countries with high TB and human immunodeficiency virus (HIV) burden [i.e. Brazil], TB/HIV co-infection accounts between 69 and 93% of TB cases admitted at an intensive care unit (ICU), usually associated with acute respiratory failure (ARF) and mechanical ventilation (MV) [3, 4]. Patients with TB admitted to the ICU have a poor prognosis and are associated with increasing costs for the health system when compared to a patient with uncomplicated TB [5, 6].

The diagnosis of pulmonary tuberculosis (PTB) in ICU settings presents challenges, mainly to obtain fast and reliable microbiological confirmation. In people living with HIV (PLWH) and critically ill patients, the diagnosis of TB may be more difficult due to the greater frequency of paucibacillary forms in advanced stages of immunodepression and develop complications related to invasive procedures [7].

Microbiological sampling from a mechanically ventilated patient will require an endotracheal aspirate (ETA), a non-directed bronchial lavage or a bronchoalveolar lavage (BAL) [7]. Although it is an easy-to-perform, low-cost and non-invasive method, the ETA has several limitations, such as low specificity and high false positive rates for the diagnosis of pulmonary infections [8].

The bronchoscopic procedures (i.e. BAL) are expensive, require qualified human resources, suitable devices (e.g. size of the bronchoscope according to the size of endotracheal tube); and it requires care with operationalization, monitoring and decontamination of equipment. Furthermore, is unavailable in many HIV and TB endemic settings [9, 10]. In addition, the BAL has been associated with several complications in patients receiving MV, especially with acute respiratory distress syndrome. Other related complications are hypoxemia, pneumothorax, cardiac arrhythmias and bleedings. Therefore, the risks/benefits of BAL should be carefully considered in the critical patient scenario prior to the application, using the technique when less invasive methods presents limitations or are not effective for diagnosis [10].

Critically ill patients are more likely to develop complications related to invasive procedures. Therefore, it is necessary that less invasive diagnostic strategies be used instead of BAL. An alternative method for obtaining respiratory samples in mechanically ventilated patients is the mini bronchoalveolar lavage (Mini-BAL); a simple, less invasive and low-cost method. It is defined as a blind and sterile technique for collection of the lower respiratory specimen by a tracheal tube (50 cm) and instillation of saline solution. The amount of instilled solution may range from 20 to 150 ml. However, small amounts of fluid (e.g. 20 ml) are commonly used [11].

Mini-BAL was first used successfully in 1987 for the diagnosis of opportunistic infection in AIDS patients [12]. It is a simple, less invasive and low-cost method. Since then, it has been used for diagnosis and prognosis of lung injury and related pulmonary diseases, including immunocompromised patients [9, 13, 14]. However, to date it has not been studied whether the Mini-BAL would be useful for the diagnosis of PTB in critically ill patients. In this study, we evaluated the performance of two methods for sample collection, Mini-BAL and ETA, for presumptive PTB critically ill patient’s diagnosis at a referral hospital for TB in PLWH located in the Brazilian Amazon.

## Methods

### Ethical issue and study population

This study was approved by the Ethics Committee at Fundação de Medicina Tropical Dr. Heitor Vieira Dourado (CEP/FMT-HVD #process: 1.531.521/2016). Signed informed consent was obtained from each participant or legal representative for the use of biological materials and publication of data. All procedures are in accordance with the Resolution 466/12 from the Brazilian Ministry of Healthy and also with the Helsinki Declaration.

**Fig. 1:**
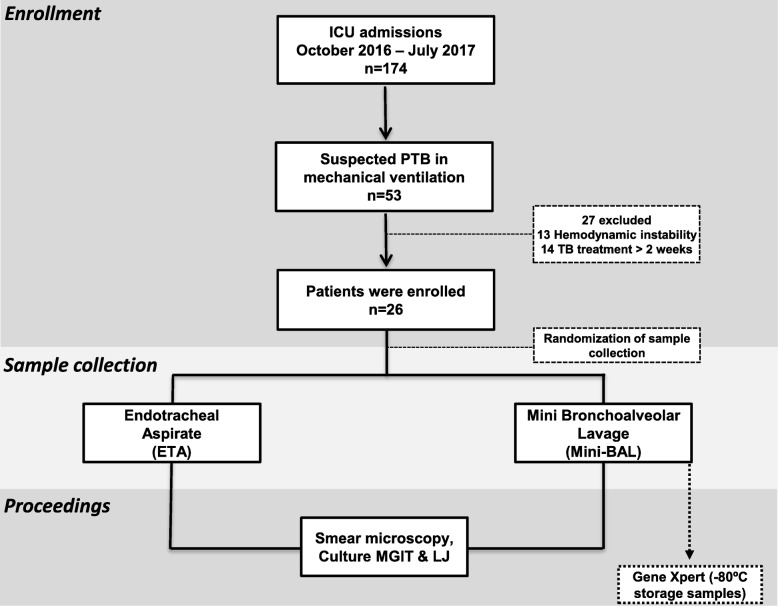
Study and laboratorial analysis flowchart of the ICU internalized patients

This prospective study (October 2016 and July 2017) involved 53 ICU internalized patients (> 18 years old), with presumptive PTB diagnosis who required MV at FMT-HVD (Fig. 1). Twenty-seven individuals were excluded for presenting complications that prevented the accomplishment of the procedures (ETA and Mini-BAL) and/or were already undergoing treatment for PTB for more than 2 weeks.

Before procedure of the collection, all vital signs were monitored, inspiratory fraction of O_2_ was increased to 1.0 and sedation was maintained with bolus of 3-5 mg intravenous midazolam.

### Sample collection randomization

Sample collection was performed using the ETA and Mini-BAL methods. Two samples were obtained from each patient and the order of collection techniques performed was randomized using the website *Random* [https://www.random.org/]. Briefly, List Randomizer function was used, which randomized the method of the according with list provided.

### ETA sample collection

ETA collection was performed using a sterile suction catheter of size 12 French (Fr) introduced through the endotracheal tuber (ET) until resistance was encountered (level of the carina in the trachea), retracted approximately 2 cm and sample collected in a sterile container by suction. The samples were aspirated into a sterile polypropylene collector tube (bronco collector; Cremer®, Brazil).

### Mini-BAL sample collection

The Mini-BAL collection was performed by a sterile long suction catheter of size 12 French (Fr) inserted through the ET and blindly advanced into the distal airways till resistance is felt then the catheter was wedged in that position. 20 ml of Sodium Chloride 0.9% were instilled through the catheter and aspirate was collected in a sterile polypropylene collector tube (bronco collector; Cremer®, Brazil) container by suction [11]. After these proceedings, the probe was delicately removed using turning movements.

### Sample transport and microbiology processing

All the samples were transported to TB Laboratory at FMT-HVD within 30 min of collection. The specimens were processed according to standard sample treatment procedures to smear microscopy, liquid and solid culture [15]. Furthermore, all samples obtained through the Mini-BAL were analyzed by the molecular test Xpert® MTB/RIF (Fig. 1).

### Liquid and solid culture test

The liquid and solid culture were performed with respiratory specimens digested and decontaminated using the N-acetyl-L-cysteine-sodium hydroxide (NALC-NaOH) method [16]. Liquid culture tubes (Mycobacteria Growth Indicator Tube - MGIT) were incubated at 37 °C in the BD BACTEC MGIT 960 system® [Becton Dickinson, Franklin Lakes, NJ, USA]) instrument and monitored automatically. The solid culture tubes (Löwenstein-Jensen [LJ] medium) were incubated at 37 °C and were inspected once weekly or until *Mycobacterium* colonies were seen. The tubes were maintained until it became positive or for 42 days of maximum for negative samples. All the positive tubes were further confirmed by Ziehl–Neelsen (ZN) staining method and further confirmed by MPT64 protein specific detection immune chromatographic test (SD Bioline Kit, Standard Diagnostics, Inc., Korea) [15].

### Molecular test Xpert® MTB/RIF

Molecular test Xpert® MTB/RIF was performed with respiratory specimens digested and decontaminated using the NALC-NaOH method [16]. The specimens were processed on the day of collection and 1 mL was tested in an Xpert® MTB/RIF cartridge-based. Rapid molecular assays were performed in GeneXpert® System (Cepheid, Sunnyvale, CA, USA) and all GeneXpert® protocols were provided by Cepheid company.

### Diagnosis of PTB

Case definition of PTB was used the following criteria: person with presumptive severe TB and growth MTB in MGIT or solid LJ culture in at least one sample obtained by Mini-BAL or ETA.

### Statistical analysis

Data were stored in the Microsoft Excel software (v.2010) and statistically analyzed using the *GraphPad Prism* (v.5, San Diego, CA, USA). Descriptive analysis was performed using number (n) and percent (%) for qualitative data and median and IQR for quantitative data. Statistical Analysis were assembled to Chi-squared test was used to study association between qualitative variables and comparison between groups was performed using Mann-Whitney Test for quantitative variables. Statistical difference was considered in all cases at *p* < 0.05.

## Results

### Patients demographic and clinical data

Table 1 summarizes of the patient’s demographic and clinical data. Of the total 26 patients included, five patients the ETA was not performed due to insufficient volume for the diagnostic tests (< 1 mL), smaller than optimal volume (5-10 mL) for liquid and solid culture, beyond the Xpert® MTB/RIF. In addition, all Mini-BAL samples could be processed, significantly number higher than by ETA (*p* = 0.018). The median age was 35 years (IQR: 29–46), with a predominance of male (73%). 92% of the patients were HIV^+^, 46% were treated for PTB and 23% evolved to death (clinical outcome). The hypoxemia was observed with ETA and Mini-BAL procedures respectively in 3 (11.5%) and in 2 (7.7%) patients (data not shown).

**Table 1:** Demographic and clinical data of the of the ICU internalized patients enrolled in study

Demographic and Clinical Data	ETA^a^ (*n* = 26)	Mini-BAL^b^ (*n* = 26)
Age (years, median [IQR])	35 [29–46]	
Sex, (male/female)	19/7	
HIV^+^, n (%)	24 (92)	
TB-treatment, n (%)	12 (46)	
Outcome (death), n (%)	6 (23)	
Gold-Standard Eligible Samples, n (%)	21^c^ (81)	26 (100)

^a^Endotracheal Aspirate; ^b^Mini bronchoalveolar lavage; ^c^5 samples of ETA had volume < 1 ml

### Volume recovery in mini-BAL was superior of the ETA

**Fig. 2:**
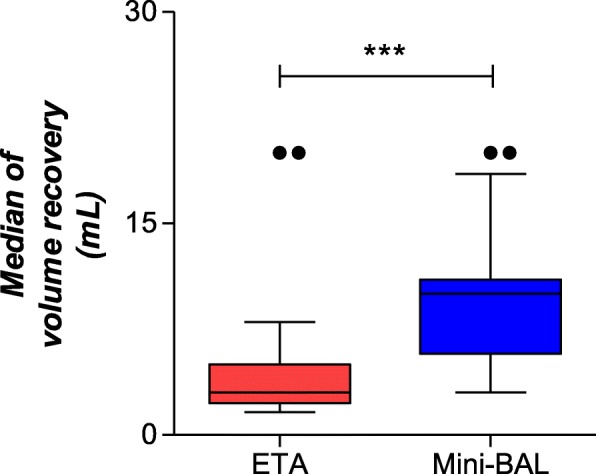
Median of Volume recovery in Mini-BAL and ETA of the ICU internalized patients

The medium sample volume was 3 ml (IQR: 2–5) for ETA and 10 ml (IQR: 6–11) for Mini-BAL (*p* < 0,0001). The Mini-BAL allowed 30% increased recovery and guaranteed sufficient sample volume for processing in all methods (Fig. 2).

### Comparison between mini-BAL and EDTA collection for diagnosis PTB

The MTB was identified in 3 (14%) and 5 (19%) samples of ETA and Mini-BAL (*p* = 0,653), respectively (Table 2). Five confirmed TB cases were observed. Two samples contaminated in solid culture with LJ medium in both techniques. ETA failed to detect TB in solid culture in two samples. Also, cases of false positive or negative in smear microscopy/MGIT/culture of ETA was identified, with one sample detected only in microscopy of ETA sample. Moreover, 5 samples obtained with the Mini-BAL had the diagnosis for MTB confirmed by the molecular method Xpert® MTB/RIF (Table 2). Furthermore, the mean time to detection (TTD) of the mini-BAL samples in the MGIT system was lower (8.2 days) but was not significantly different from the TTD of the ETA (9.0 days) (*p* = 0,134) (data not shown).

**Table 2:** Results of diagnosis methods used for presumptive PTB in critically ill patients of the ICU internalized

Diagnosis Methods Description	Collection Techniques		
	ETA (*n* = 21^a^)	mini-BAL (*n* = 26)	*P value*
Smear microscopy-positive, n (%)	6 (29)	5 (19)	0.451
Culture MGIT-positive, n (%)	4 (21)	5 (19)	0.987
Culture LJ-positive, n (%)	3 (16)	5 (19)	0.653
Xpert MTB/RIF®-positive, n (%)	–	5 (19)	–

^a^5 samples of ETA had volume < 1 ml

## Discussion

The detection of MTB in the ICU presents several challenges, mainly associated to the clinical state of the patient. The presence of HIV infection further aggravates this scenario, requiring a reliable collection method, with better performance in the microbiological/molecular techniques to be performed. The diagnosis of TB in HIV infected individuals may be more difficult due to the greater frequency of paucibacillary forms in advanced stages of immunodepression [9, 10]. Our results demonstrated that the Mini-BAL proved to be a viable alternative, since these critically ill and often immunocompromised patients are more likely to develop complications related to invasive procedures.

Patients with TB required ICU care in worldwide, may have high rates of co-morbidities related complications this site. In other ICU patients, diagnostic of TB may be an incidental co-morbid finding as previously undiagnosed sub clinical disease which only manifests during hospitalization [6, 17]. Mortality is high for patients with active TB and respiratory failure, mainly associated with delay in the TB diagnosis. As shown in our report (23%), other studies have shown similar data, ranging between 25 and 65% mortality [4, 18].

ICU patients present particularities, especially in cases presumptive PTB critical ill [6]. The sampling collections with ETA technique in these patients may sometimes be restricted by persistent severe hypoxemia or cardiovascular instability, presence of small endotracheal tubes and/or the unavailability of a bronchoscopist [14]. Thus, the diagnosis of cases presumptive PTB critical with Mini-BAL seems to be a viable alternative, since this method is less-invasive, easily performed at the bedside method and offering quality samples for microbiological identification, especially in middle- and low-income countries.

Although insufficient data regarding the utility of Mini-BAL to TB diagnosis, some studies have applied the technique for the diagnosis of ventilator-associated pneumonia, including in immunocompromised patients [9, 19–22]. In addition, Mini-BAL was used for the biomarker identification in patients with lung injury [13], evaluated inflammation in patients with acute hypoxemic respiratory failure [14], cytological diagnosis of interstitial disorders with ARF and MV [23], concentration and absorption of antibiotics [24] and the safety and diagnostic accuracy of transbronchial biopsy [25]. Possible complications such as hypoxemia, arrhythmia and bleeding were also evaluated in each technique.

In our study, Mini-BAL allowing the recovery of median 10 mL of respiratory sample (IQR 5-10 mL), significantly more than the volume recovered (*p* < 0,0001), with non-inferior results to ETA for MTB detection. In addition, the larger volume of the samples obtained with the Mini-BAL (10 mL) enabled the Xpert® MTB/RIF molecular test for diagnosis of TB. An adequate sample volume may be critical for the diagnosis of TB. For example, a sample volume of 6 mL or more of cerebrospinal fluid (CSF) was associated with higher MTB detection in patients with suspected tuberculous meningitis using Xpert® MTB/RIF Ultra or solid culture as a diagnostic method [26]. For PTB, greater sputum volume may have a positive impact on detection of *Mycobacterium tuberculosis* by Xpert® MTB/RIF in smear-negative patients [27].

According to Practice Guideline for clinical microbiology laboratories that addresses requirements pertaining to laboratory testing for mycobacteria and aspects of acceptable specimens and rejection criteria, an optimal volume for samples of the respiratory tract between 5 and 10 ml is recommended [28]. Therefore, 7 (27%) ETA samples were considered inadequate because they presented insufficient volume (< 5 ml) and five (19%) ETA samples were excluded to insufficient volume for the diagnostic tests (Table 1).

To our knowledge, this is the first study to show that samples obtained by the Mini-BAL are viable for the molecular diagnosis of TB (i.e. Xpert® MTB/RIF) in critically ill patients. All samples obtained by the Mini-BAL could be processed in the Xpert machine, without occurrence of errors or indeterminate results.

Our study is limited due to small sample size. However, the techniques were randomized in each patient to mitigate possible bias, may become the Mini-BAL an acceptable alternative to tracheal aspiration for the evaluation of suspected tuberculosis in critically ill patients admitted at an intensive care unit (ICU).

## Conclusion

In summary, Mini-BAL presented similar results for the diagnosis of PTB when compared to ETA. Furthermore, respiratory samples obtained by Mini-BAL were able to be tested for *M. tuberculosis* detection, as well as, greater recovery of specimens, in critically ill patients with presumptive PTB, showing an acceptable alternative to ETA in this population. Future research is needed to validate these results, establish their general applicability, and determine the influence of the laboratorial diagnostic in clinic and patient follow-up.

## Data Availability

The data sets generated during the current study are available from the corresponding author on reasonable request.

## Abbreviations

ARF: Acute respiratory failure
ETA: Endotracheal aspirate
HIV: Human immunodeficiency virus
ICU: Intensive care unit
LJ: Lowenstein Jensen
MGIT: Mycobacteria growth indicator tube
Mini-BAL: Mini bronchoalveolar lavage
MTB: *Mycobacterium tuberculosis*
MV: Mechanical ventilation
PTB: Pulmonary tuberculosis
TB: Tuberculosis
TTD: Time to detection
